# Influence of Nitrogen Doping on Device Operation for TiO_2_-Based Solid-State Dye-Sensitized Solar Cells: Photo-Physics from Materials to Devices

**DOI:** 10.3390/nano6030035

**Published:** 2016-02-23

**Authors:** Jin Wang, Kosti Tapio, Aurélie Habert, Sebastien Sorgues, Christophe Colbeau-Justin, Bernard Ratier, Monica Scarisoreanu, Jussi Toppari, Nathalie Herlin-Boime, Johann Bouclé

**Affiliations:** 1 IRAMIS/NIMBE/LEDNA, UMR 3685, CEA Saclay, 91191 Gif sur Yvette, France; 516208050@qq.com (J.W.); aurelie.habert@cea.fr (A.H.); 2 Nanoscience Center, Department of Physics, University of Jyväskylä, P.O. Box 35, 40014 Jyväskylä, Finland; kosti.t.o.tapio@jyu.fi (K.T.); j.jussi.toppari@jyu.fi (J.T.); 3 Laboratoire de Chimie Physique, UMR8000, Université Paris-Sud, 91405 Orsay, France; sebastien.sorgues@u-psud.fr (S.S.); christophe.colbeau-justin@u-psud.fr (C.C.-J.); 4 XLIM UMR 7252, Université de Limoges/CNRS, 87060 Limoges Cedex, France; bernard.ratier@unilim.fr; 5 National Institute for Lasers Plasma and Radiation Physics, P.O. Box MG 36, R-077125 Bucharest, Romania; monica.scarisoreanu@inflpr.ro

**Keywords:** solid-state dye-sensitized solar cells, TiO_2_, nitrogen doping, photo-physics, photo-response, spiro-OMeTAD

## Abstract

Solid-state dye-sensitized solar cells (ssDSSC) constitute a major approach to photovoltaic energy conversion with efficiencies over 8% reported thanks to the rational design of efficient porous metal oxide electrodes, organic chromophores, and hole transporters. Among the various strategies used to push the performance ahead, doping of the nanocrystalline titanium dioxide (TiO_2_) electrode is regularly proposed to extend the photo-activity of the materials into the visible range. However, although various beneficial effects for device performance have been observed in the literature, they remain strongly dependent on the method used for the production of the metal oxide, and the influence of nitrogen atoms on charge kinetics remains unclear. To shed light on this open question, we synthesized a set of N-doped TiO_2_ nanopowders with various nitrogen contents, and exploited them for the fabrication of ssDSSC. Particularly, we carefully analyzed the localization of the dopants using X-ray photo-electron spectroscopy (XPS) and monitored their influence on the photo-induced charge kinetics probed both at the material and device levels. We demonstrate a strong correlation between the kinetics of photo-induced charge carriers probed both at the level of the nanopowders and at the level of working solar cells, illustrating a direct transposition of the photo-physic properties from materials to devices.

## 1. Introduction

Since the pioneering work of Bach *et al.* in 1998 [1], solid-state dye-sensitized solar cells (ssDSSC) based on the organic molecular glass 2’,7,7’-Tetrakis-(*N*,*N*-di-4-methoxy phenylamino)-9,9’-spirobifluorene (spiro-OMeTAD) as p-type solid-state electrolyte have demonstrated constant performance improvement, thanks to the rational engineering of organic sensitizers and doping strategies. In particular, doping of hole-transporting materials (HTM) by cobalt complexes [2] or organic compounds such as 1,1,2,2-tetrachloroethan (TeCA) [3] have led to power conversion efficiencies over 7% under standard illumination conditions, illustrating the relevance of hybrid solid-state approaches for solar energy conversion. Improved spectral coverage of the solar spectrum as well as enhanced light harvesting efficiencies are now achieved by exploiting various metal-free organic dyes [4], such as porphyrin [5] or arylamine derivatives [6]. More recently, and apart from perovskite solar cells [7,8], which are not the primary topic of this article, ssDSSC based on TiO_2_ porous electrodes have received additional benefits from the intensive developments made on HTMs [9,10,11]. Consequently, efficiencies over 8% were demonstrated using p-type perovskite materials [12,13] or Copper phenanthroline complexes [14] such as HTM.

In a typical device, the TiO_2_ porous electrode acts simultaneously as a high specific area substrate for dye adsorption, and as the electron transporting material [15,16]. The metal oxide photo-electrode is therefore crucial for both light absorption and current generation. Several approaches have been proposed to improve the charge generation and collection efficiencies of nanostructured metal oxide electrodes using alternative electrode morphologies, such as nanorods or hierarchical structures [17]; substitution of TiO_2_ by other metal oxides such as ZnO or SnO_2_ [18]; deposition of insulating oxide shell on TiO_2_ [19,20]; insertion of metallic (Au, Ag, …) [21,22] or non-metallic (S, N, *etc*.) [23,24,25,26] elements. Among all of these strategies, the doping of TiO_2_ materials by nitrogen can exploit several effects, which can positively affect device performance. Asahi *et al.* reported in 2001 that, compared to pure TiO_2_, N-doped TiO_2_ exhibits a broader absorption range that extends into the visible up to 500 nm [27]. Since then, some attention has been paid to the origin of this additional absorption feature [28]. In a theoretical approach [29,30], Di Valentin *et al.* pointed out that if N atoms are in substitutional positions, N2p states can lie 0.13 eV above the top of the valence band of TiO_2_. Moreover, if N is found in interstitial positions or at the surface of the nanoparticles, NO species can be formed and introduce π*-NO-states into the band gap (0.73 eV above the top of the valence band). Asahi *et al.* investigated the effects of different nitrification conditions, and found out that substitutional N can be chemically more stable in the presence of oxygen vacancies [31]. Therefore, benefits induced by the N-doping rapidly raised the interest of researchers in the fields of photo-catalysis and photovoltaics. 

In the field of hybrid solar cells, Ma *et al.* demonstrated an N-TiO_2_ based liquid DSSC presenting an improved efficiency and stability than that prepared from a pristine TiO_2_ electrode [24]. An enhancement of the incident photon to charge carrier efficiency (IPCE) was observed within the 380–520 nm range, related to the contribution of nitrogen to the absorption of N-TiO_2_ powders. Tian *et al.* analyzed the electron lifetime in the sol-gel synthesized N-TiO_2_ solar cells and found that the formation of O-Ti-N in the TiO_2_ lattice could retard charge recombination reactions at the TiO_2_ electrode/electrolyte interface [32]. In addition, the insertion of N atoms can alter the Fermi level of electrons in the oxide [33], potentially leading to a slight increase of the open-circuit voltage of the cell, as well as of the overall device efficiency. However, most of the studies reported on N-doping were carried out on liquid DSSC. In our previous study [34], we reported on solid-state DSSCs based on N-doped TiO_2_ electrodes processed from nanocrystals synthesized by laser pyrolysis. We provided evidence of a significant contribution from the N-doped electrode to the generation of charge carriers, as a secondary current generation pathway. Although recent studies based on quasi-solid state DSSC devices demonstrated similar trends [35], no clear correlation between material properties and device performance were drawn.

In this work, we systematically report on the influence of nitrogen doping on ssDSSC device operation and photo-physics properties. To this end, we synthesized a set of TiO_2_ nano-particles doped with different levels of nitrogen. Using complementary characterization techniques, both at the material and device levels, we focus on the impact of the presence of N atoms on the photo-generated charges in the metal oxide, and discuss its implications on the photovoltaic performance of the cells. X-ray photoelectron spectroscopy (XPS) is exploited to monitor the exact location of N atoms in the TiO_2_ crystalline sites, while time-resolved microwave conductivity (TRMC) is used to assess its impact on photo-generated charge dynamics. Photo-conductivity measurements performed on test devices based on un-doped and N-doped porous TiO_2_ electrodes are finally discussed with regard to charge recombination kinetics measured on full devices using transient photo-voltage. Considering the different treatments applied to the powders for the preparation of porous electrodes suitable for device testing, such methodology is particularly relevant to reveal the influence of the nitrogen doping both in the starting powders and in the final solar cells.

## 2. Results

### 2.1. Properties of the Starting Powders

**Figure 1:**
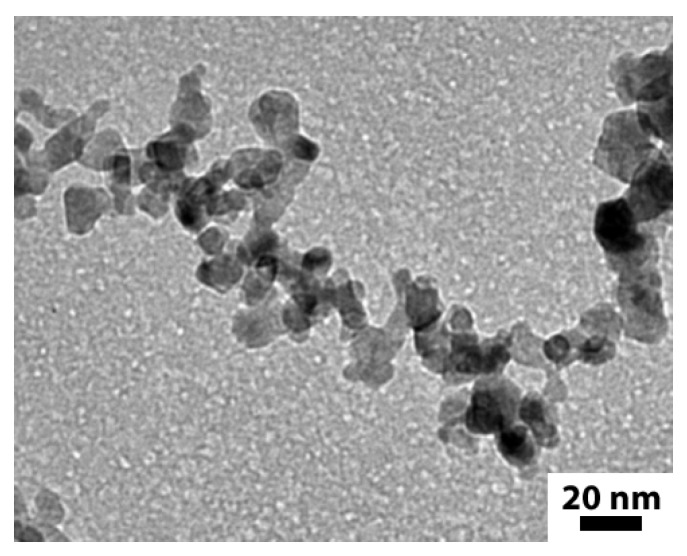
Transmission electron microscopy (TEM) image of a typical N-doped TiO_2_ powder (after annealing treatment in air). The nitrogen content is 0.2 wt % in this case.

Laser pyrolysis was used to synthesize a set of N-doped TiO_2_ nanopowders [36]. This technique is an efficient method for the production of well-controlled nanocrystals with tunable properties, well-adapted for the photovoltaic application [37,38]. Here, the precursor mixture included ammonia as the source of N atoms. More details on the experimental conditions used for the synthesis of the powders can be found in Supplementary materials (Table S1), which also summarizes the chemical composition of the samples. In all cases, the as-synthesized nanopowders contain free carbon phases easily removed by thermal annealing under air. This treatment, which does not alter the main physical properties of the samples [36], was therefore applied to all powders considered in the following parts of this article. Figure 1 presents the transmission electron microscopy (TEM) image of a typical TiO_2_ powders (doped with nitrogen in this case).

We observed a typical “chain-like” morphology of nano-scaled grains (mean diameter within 10 to 20 nm), typical of gas phase synthesis methods.

Table 1 summarizes the main physical parameters of the powders synthesized in this work, including nitrogen content, crystalline phase and crystallite diameter extracted from X-ray diffraction (XRD), as well as specific area and mean particle diameter estimated from the BET (Brunauer, Emmett, and Teller) method. The Spurr and Scherrer equations were used on the XRD patterns (Supplementary materials, Figure S1) to extract the Anatase to rutile crystalline fraction and the mean crystal diameter [39,40].

The N content in the final powders is directly driven by the level of ammonia in the precursor mixture (see Supplementary materials Table S1). The sizes estimated by BET are slightly larger than those extracted from the XRD analysis, revealing the presence of amorphous regions in the powders, or a slight particle agglomeration [36]. Both the crystalline phase and particle diameter are only slightly dependent on the doping level, leading to five powders with rather comparable morphologic features.

**Figure 2:**
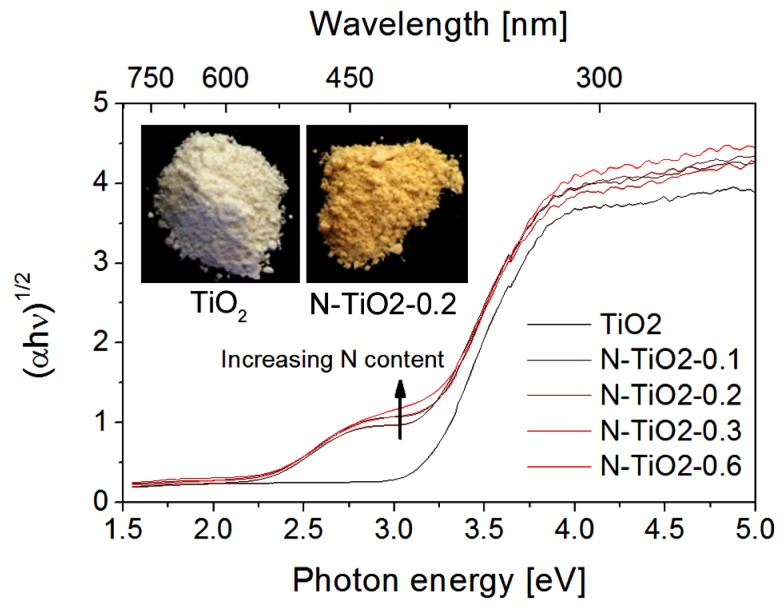
Optical data extracted from diffuse reflectance measurements on the TiO_2_ and N-doped TiO_2_ powders. The inset presents pictures of the N-TiO_2_-0.2 sample (N content of 0.2 wt %) compared to the TiO_2_ reference sample.

Regarding their optical properties, N-doped TiO_2_ powders exhibit a dominant yellow color (see inset of Figure 2), indicating a shift in their absorption threshold towards the visible range. Figure 2 shows the Kubelka–Munk function of the powders calculated from diffused reflectance measurements. Comparing to pure titania, N-doped TiO_2_ powders exhibit an additional absorption band between 370 and 550 nm. This optical feature, which was discussed in previous reports [24,34], can be assigned to the influence of nitrogen on the energetic level of TiO_2_, through a mixing of N and O 2p states [27]. We observe that the intensity of this additional absorption band increases with the N doping level.

**Figure 3:**
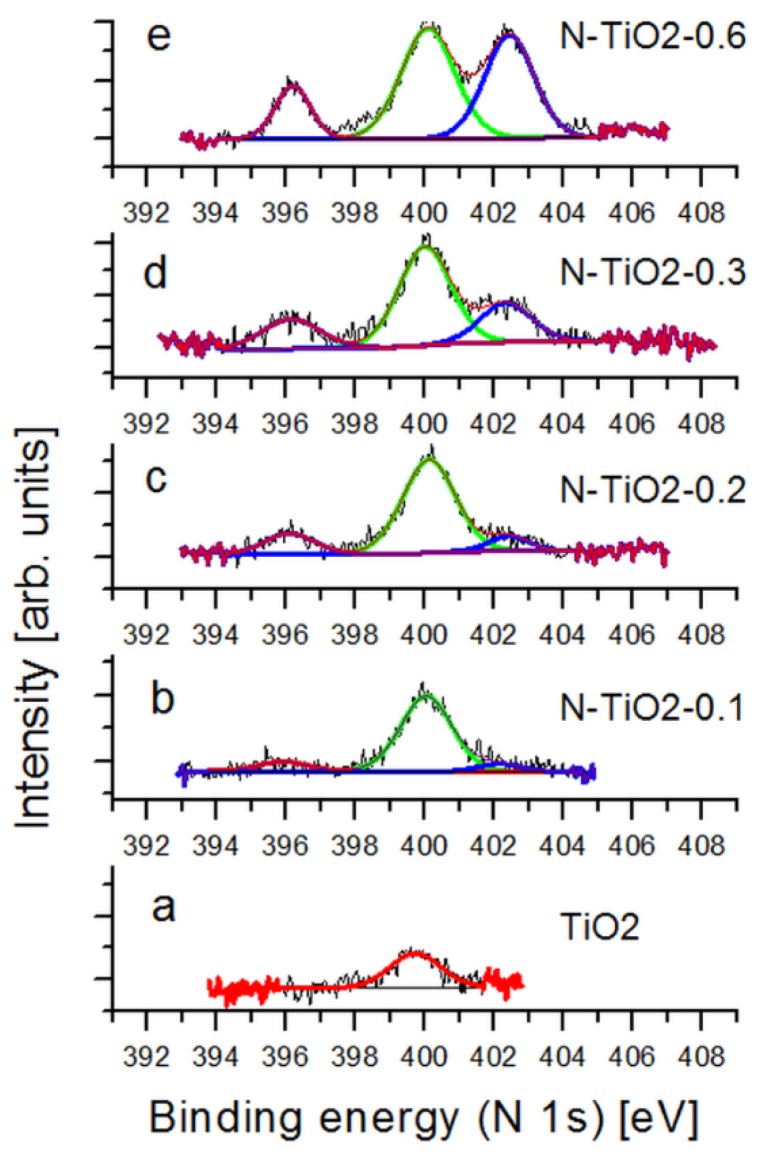
XPS spectra (N 1s) of the undoped and N-doped TiO_2_ powders as a function of N content (from 0 to 0.6 wt %).

A more detailed picture of the local environment of nitrogen atoms in the metal oxide structure is drawn from X-ray photoelectron spectroscopy (XPS) applied on the powders. Data associated with annealed powders are presented in Figure 3, together with their deconvolution, while data associated with as-prepared samples are presented in Supplementary materials (Figures S2 and S3, respectively).

Concerning the pure TiO_2_ sample, the only peak appearing at 399.7 eV in the spectra, both before and after annealing (see Supplementary materials), can be attributed to N_2_ species chemically adsorbed on the TiO_2_ surface, which is a feature being often observed in the literature [41]. The situation is more complex in the case of the N-doped powders. Three main contributions centered at 396.2 eV, 399.9 eV, and 402.2 eV are observed, and their exact assignment is still under debate in the literature [27,32,41,42]. The peak at 396.2 eV is usually attributed to Ti-N bonds and it thus implies that N atoms are situated at substitutional sites in the TiO_2_ lattice [24]. In most of the cases, the peak at 399.9 eV is assigned to O-Ti-N bonds—in other words, to interstitial nitrogen atoms [29,32,43,44]. This assignment is consistent with the high electronegativity of oxygen, which reduces the electron density on nitrogen compared to Ti-N. As a result, the binding energy of O-Ti-N is slightly larger than that of Ti-N. However, several groups associate this peak with NO in interstitial sites or NO_2_ in substitutional sites [31]. We reckon that part of this peak can also result from chemically adsorbed N_2_ species on the TiO_2_ surface, like in undoped TiO_2_. However, comparing the absolute intensities of the signals, we can safely conclude that the peak at 399.9 eV in our N-TiO_2_ samples can be assigned to interstitial nitrogen. The last peak at 402.2 eV is often observed in the literature [24]. Asahi *et al.* [27], as well as Tian *et al.* [32], assigned it to atomically adsorbed N species. Therefore, we associate this peak as the signature of nitrogen present at the surface of the TiO_2_ particles. These surface N atoms should however be quite different than the adsorbed N_2_ species observed on the surface of the pure TiO_2_ samples (Figure 3a), as the associated binding energies are quite dispersed on all N-doped spectra (Figure 3b–e). Considering the nature of our samples, this third feature is likely to be associated to NO or NO_2_ groups on the particle surface. Table 2 summarizes the relative contributions of the three features associated to nitrogen in the doped samples (see the Supplementary materials for data associated with non-annealed samples, Table S2).

Comparing the relative contributions of the different peaks in the as-prepared and annealed powders, we observe a significant decrease of the intensity of the 396.2 eV peak with annealing (Table S2 and Table 2), which indicates a significant oxidation of substitutional Nitrogen. DFT calculations performed by Di Valentin *et al.* [40] show that the transition from substitutional to interstitial N is an exothermic process. Under oxygen-poor conditions, substitutional N position is favored. This is typically our case during particle growth, as powders are synthesized under inert or reducing conditions. In the opposite case, the annealing treatment performed at 400 °C in the presence of oxygen results in the rapid oxidation of substitutional nitrogen atoms. Thus, for low nitrogen content, most of the N atoms are located in interstitial positions for annealed powders (about 89% of all nitrogen atoms for sample doped at 0.1 wt %). However, for higher doping levels, not only the percentage of substitutional N increases, but a strong increase of the contribution of surface nitrogen is also observed. For comparison, Wang *et al.* reported that most of the N atoms were located only in interstitial sites for TiO_2_ doped with 1.53 atom % of nitrogen [44]. In our case, because substitutional N atoms are always present in as-prepared samples, these types of N atoms are already present at low doping levels. In particular, our analysis suggests that the thermal treatment leads to an overall decrease of the substitutional N present in as-prepared samples (due to the oxygen-free synthesis conditions) and to a migration of the N atoms to the surface of the particles, especially at high doping levels. This feature is an important drawback of nitrogen doping, as free charge generation and current collection is drastically limited by surface states in DSSC and ssDSSC. 

### 2.2. Photovoltaic Performance of ssDSSC Based on N-Doped TiO_2_ Electrodes

**Figure 4:**
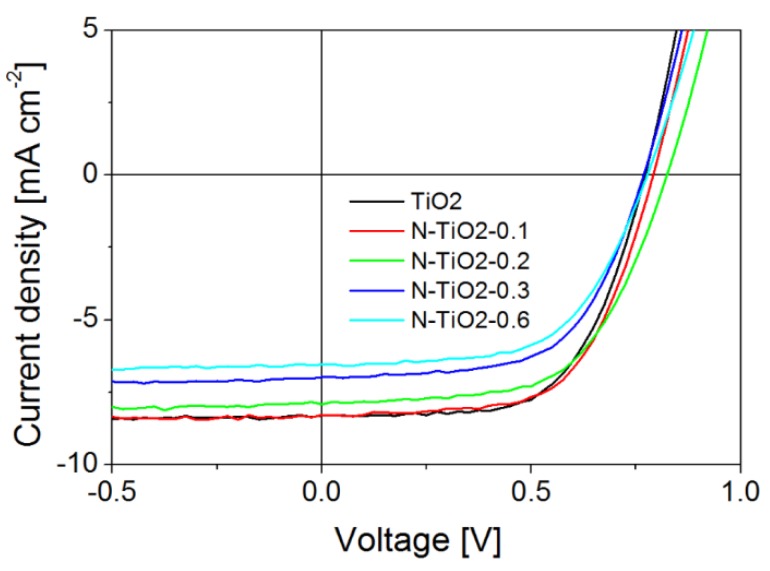
Current density/voltage characteristics of ssDSSC solar cells under standard illumination conditions (AM1.5G, 100 mW°C·cm^−2^) for the different N contents.

Several independent sets of solid-state dye-sensitized solar cells (ssDSSC) were prepared from the un-doped and doped metal oxide nanopowders following procedures already described [34,37]. Briefly, a metal oxide paste is initially formulated and used to deposit the electrode on FTO/compact TiO_2_ substrates. After sintering, porous electrodes of around two microns thick are obtained, which are further sensitized by the D102 indoline dye and infiltrated by the reference spiro-OMeTAD molecular glass acting as HTM. The conventional dopants (lithium salt and *tert*-butylpyridine) are used in our case. SEM cross sections of the infiltrated D102-sensitized electrodes are presented in Supplementary materials for the undoped and N-doped electrodes (Figure S4). In all cases, the solid-state electrolyte is clearly visible down to the bottom of the electrode. This observation is consistent with the morphologies of the starting nanopowders. Rather similar particle morphologies result in similar porous electrode morphology and, keeping all other parameters equal (nature of the dye, HTM concentration, and infiltration parameters), lead to comparable pore filling fractions. The absorption coefficient of the dye-sensitized electrodes, before HTM infiltration (Supplementary materials, Figure S5), does not clearly reveal any additional band associated to N-doping. However, a slight increase in absorption with increasing N content is observed over the entire wavelength range, with a more pronounced effect in the the 400–550 nm region. This region covers both the absorption band related to nitrogen doping in TiO_2_ and the absorption band of the D102 dye, usually centered at 480 nm. It is thus reasonable to suggest that nitrogen doping is likely to slightly contribute to this increase of absorption. However, we believe that the main effect of doping is related to a better sensitization of the electrodes, as previously reported [24,34]. A better dye grafting may also result from a change in surface potential induced by doping [45]. The electrical characteristics of the solar cells under illumination (AM1.5G, 100 mW·cm^−2^) are presented in Figure 4, while Table 3 summarizes the corresponding photovoltaic parameters (the electrical characteristics in the dark are presented in Figure S6). 

Let us note here that our reference device based on pure TiO_2_ shows state-of-the-art performance considering the material used [37,46]. A slight improvement of solar cell efficiency is evident for low nitrogen content (up to 0.1 wt %) compared to the pure TiO_2_ electrode, before a drastic decrease in performance at higher doping levels. Although the beneficial influence of nitrogen at low doping levels on the overall power conversion efficiency seems not so clear, the trend is unambiguously confirmed through several independent set of devices (not shown here), and is consistent with our preliminary report [34]. The incident photon to charge carrier efficiency (IPCE, or external quantum efficiency EQE) spectra of the cells (Figure S7, Supplementary materials) still especially exhibit an increase of photocurrent generation in the 400–500 nm region that can be related to nitrogen doping. We also note a significant decrease of IPCE as a function of doping level in the 550–650 nm region. This drop in photocurrent generation efficiency is related to the existence of surface-related electronic features, as revealed by photoluminescence spectroscopy on the nanopowders (Figure S8, Supplementary materials). A typical emission at 2.03 eV, associated with radiative recombination of self-trapped excitons at the particle surface, is observed for all powders [38]. This emission is found more pronounced in the presence of N atoms (especially when they are located at the particle surface), and results in a rapid recombination of photo-generated excitons following excitation in this spectral range. This decrease of IPCE in the 550–600 nm region is in fact counterbalancing the relative increase around 450 nm, leading to lower photocurrent as the doping level increases. 

Going back to device performance, when the doping level reaches 0.2 to 0.3 wt %, device performance starts to significantly drop. Xie *et al.* also reported this trend, although no threshold was clearly pointed out [47]. For liquid cells, Guo *et al.* observed an optimal doping level of about 0.4 atom % [45], which is consistent with our data if we consider that our best performing device is associated with a nitrogen content of 0.1 wt %.

If we carefully check the photovoltaic parameters of the cells, the open circuit voltage of the N-doped devices is slightly improved compared to pure TiO_2_, as usually observed in the literature [24,48]. This increase is likely to be due to a slight shift of the quasi-Fermi level of electrons in N-TiO_2_ [29], especially if we consider that nitrogen is more concentrated at the particle surface. The short-circuit current density is at the maximum for both the un-doped device and for the one based on the lowest Nitrogen content (N-TiO_2_-0.1), before decreasing for a higher doping level. In most of the cases, larger currents are evident for N-doped devices when the doping level is near this optimum concentration. This improved photocurrent, also observed in our preliminary study [34], was attributed to a beneficial contribution of nitrogen on the optical absorption of the electrode in the visible range [28,49]. Moreover, in the studies reporting significant increases in photocurrents and efficiency with doping, N atoms were mainly detected in substitutional or interstitial positions [48,50]. Smaller currents for N-doped electrodes compared to un-doped reference cells have also been reported in some cases [32]. The next sections of this article will focus on the elucidation of the relation between charge kinetics and nitrogen location going from materials to devices using techniques adapted to each scale. 

### 2.3. Charge Kinetics Probed by Transient Photo-Voltage at the Device Level

**Figure 5:**
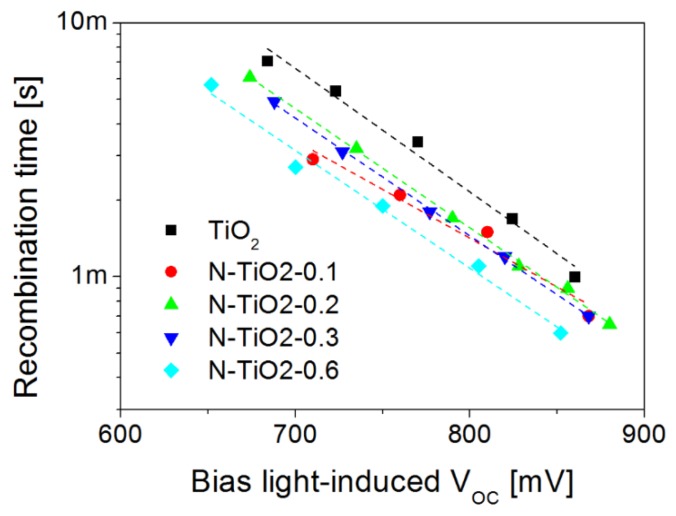
Recombination kinetics of ssDSSC probed by transient photo-voltage decay measurements, as a function of doping level.

To get a better insight into the exact influence of nitrogen on charge kinetics in ssDSSC devices, we performed transient photo-voltage measurements under working conditions, as a function of the incident light intensities. Under open circuit conditions, the transient photo-generated charges can decay only through recombination at the TiO_2_-dye-HTM interface, as no carrier can be extracted in the external circuit. Figure 5 presents the corresponding recombination time for ssDSSC based on pure and N-doped TiO_2_ electrodes. 

Clearly, the charge lifetime decreases in all N-doped devices. Furthermore, recombination seems to become faster as the nitrogen content increases, especially under high light intensities (when the bias-light induced open-circuit voltage of the cells exceeds 800 mV). This initial observation indicates that N-doping is responsible for accelerated charge recombination in the devices, especially under standard illumination conditions. Our observation is opposite to that of Tian *et al.* who reported that N-doped TiO_2_ can retard charge recombination due to the presence of O-Ti-N bonds [32]. However, faster recombination has been observed for N-doped electrodes elaborated from particles with large diameters (>20 nm) [49,51,52]. Such a phenomenon being unlikely in our case (see Table 1), this decrease in charge lifetime seems to be a direct consequence of the presence of N dopants in our TiO_2_ materials. In particular, the preferential location of nitrogen atoms at the particle surface for high doping levels is consistent with shorter charge carrier lifetimes and lower performance. However, charge kinetics probed at the level of working devices is potentially strongly affected by the various processing steps used for cell fabrication, including sintering steps at high temperature which may significantly alter the nitrogen distribution. Therefore, the next section focuses on the characterization of charge dynamics at the material level. 

### 2.4. Charge Kinetics Probed by Transient Techniques at the Material Level

**Figure 6:**
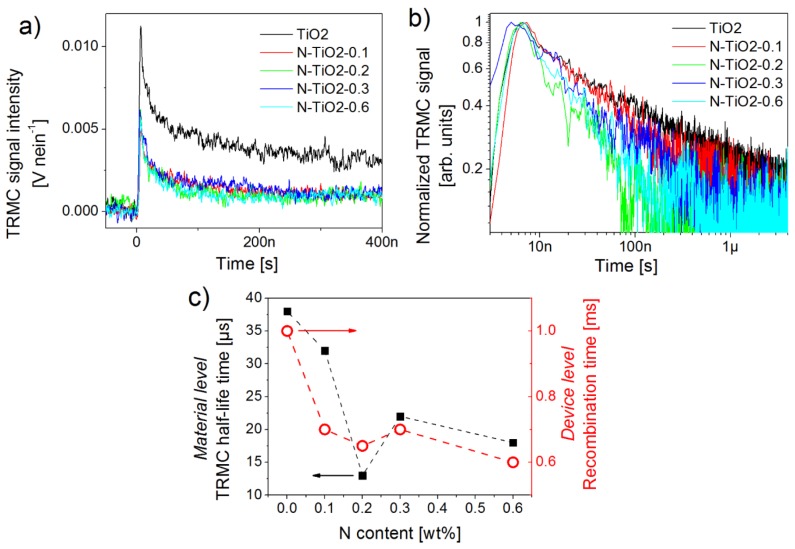
(**a**) absolute and (**b**) normalized time-resolved microwave conductivity (TRMC) signals of pure and N-doped TiO_2_ powders with light excitation at 355 nm for the different N-doping levels; (**c**) TRMC decay half-times (black squares) measured at the material level on the TiO_2_ and N-TiO_2_ powders, and compared to recombination times extracted from transient photo-voltage at the device level.

In order to understand the role played by nitrogen atoms on charge generation, time-resolved microwave conductivity (TRMC) measurements were performed on the starting TiO_2_ and N-TiO_2_ powders. This technique, briefly described in Supplementary materials, is not a common tool of photo-physicists and photo-chemists. It was however successfully used to analyze charge kinetics of metal oxide materials for photo-catalysis [53,54,55] and photovoltaics [56,57,58]. It appears particularly well suited to investigate the influence of doping on the photo-conductivity properties of TiO_2_ in the context of this study [59]. Figure 6 presents the absolute and normalized TRMC signals for the pure and N-doped TiO_2_ before and after light excitation at 355 nm. In order to remove the effect of the number of photons, all the TRMC signals presented in this work are divided by the number of photons in nanoEinstein (nein), corresponding to the number of nanomoles of photons.

The maximum intensity of the TRMC signal depends on three factors: the absorption coefficient of the material at the excitation wavelength; the interaction between the microwave electronic field and the materials, *i.e.*, the dielectric constant of the material; the recombination rate of electrons and holes during the laser pulse. Considering that the absorption coefficient at 355 nm of both un-doped and doped TiO_2_ is mainly driven by transitions from O 2p to Ti 3d orbitals, we safely assume a similar absorption coefficient for all samples. We also assume a rather similar dielectric constant for the powders, as they present comparable crystalline structure and morphology (see Table 1), assuming low doping levels (always below 1 wt %). In these conditions, the strong decrease of TRMC signal with doping is consistent with additional recombination of free charge carriers during the laser pulse. Such observation is consistent with a previous report on N-doped titania materials [59], as well as with our transient photo-voltage analysis performed at the level of working devices (Figure 5). Accordingly, faster TRMC decay rates are observed with increasing N content in the nanopowders compared to the reference (Figure 6b). In addition, and except for sample N-TiO_2_-0.2, the decay rate is faster for large nitrogen content (Figure 6c). This is quite expected as when N atoms are inserted into the TiO_2_ lattice, additional defects, such as oxygen vacancies, are spontaneously generated to ensure the global electric neutrality of the system. The photo-generated electrons are therefore more likely to be trapped in such defects, with a probability following in principle the nitrogen content. In our previous work, DFT calculations confirmed the occurrence of oxygen vacancies induced by the presence of nitrogen dopants [34]. Such processes have also been reported by other groups [59]. Finally, the extracted TRMC half-life time shows a strong correlation with the recombination kinetics measured on devices by transient photo-voltage decays (Figure 6c). This correlation, which was also confirmed through charge kinetics extracted from impedance spectroscopy applied on the same devices (not shown here), shows, in this particular case, the relevance for a multi-scale photo-physical approach from materials to devices. Our observations suggest that the features introduced by nitrogen doping are preserved during device fabrication, and that the dynamics of photo-generated charge carriers probed in the TiO_2_ nanopowders still mainly drives device performance under simulated sunlight. 

**Figure 7:**
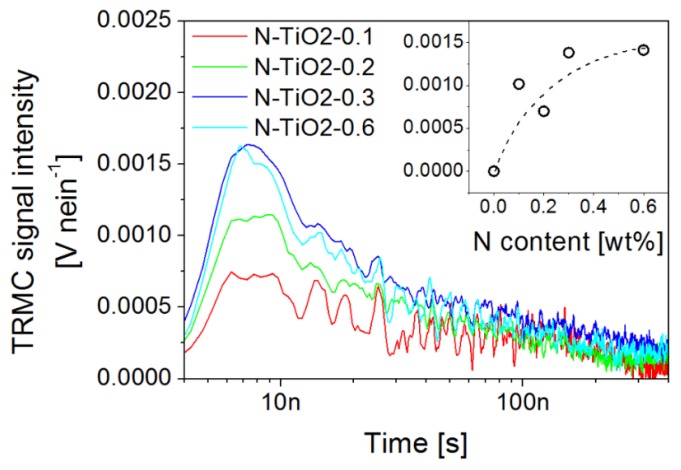
TRMC signals associated with an excitation at 450 nm, for N-doped powder samples. The inset shows the maximum TRMC signal amplitude (recorded after 9 ns in all cases) as a function of N content in the nanopowders.

Considering the photo-activity of the N-doped samples in the visible region, we now give a better look at the influence of nitrogen on charge kinetics probed by TRMC using an excitation in the visible range. First, no TRMC signal is evidenced for the un-doped TiO_2_ powder, in accordance with a flat absorption in this region. However, significant TRMC signals are recorded for the N-doped nanopowders, which is consistent with their optical absorption (Figure 2). No significant differences in the TRMC decay profiles of doped samples are, however, observed by exciting them either at 355, 420, 450, or 480 nm (see Supplementary materials, Figure S9 corresponding to sample N-TiO_2_-0.6). Similar reports were made in the literature [59], which are associated with the fact that both the O 2p to Ti 3d and the N 2p to Ti 3d transitions, induced through UV and visible excitation, respectively, show a similar final state. By fixing the excitation to 450 nm, the amplitudes of the TRMC signals increase with the nitrogen content up to 0.6 wt % in the powder (Figure 7), indicating that the photo-conductivity of the samples increases with the nitrogen content during the first 100 ns. This observation is consistent with the creation of a larger amount of electron-hole pairs for high doping levels. Considering our previous conclusions, this result suggests that a competition between charge transport and recombination is occurring in doped samples. In order to better interpret these effects, we finally analyze more carefully the electrical photo-conductivity of porous TiO_2_ electrodes processed from the doped and un-doped nanopowders, *i.e.*, an intermediate elaboration level between the powder and the device. 

### 2.5. Photo-Conductivity Measurements on Porous Electrodes

**Figure 8:**
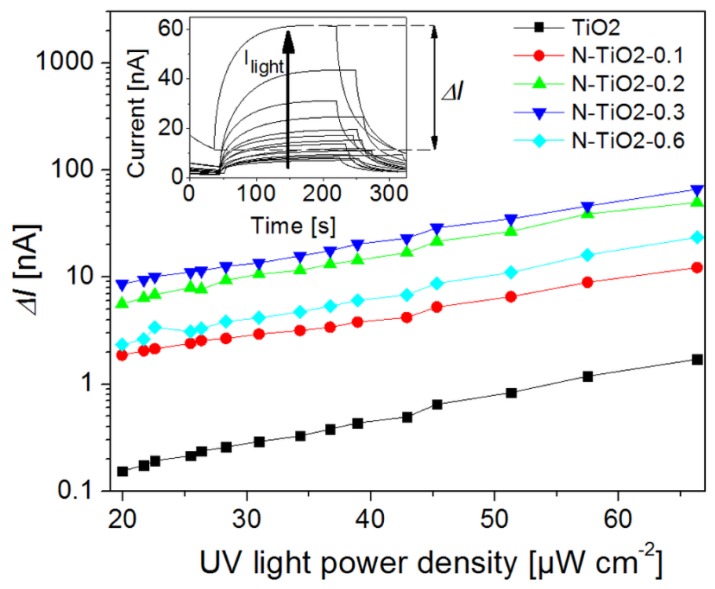
Photo-current response ΔI(t) under UV excitation of porous TiO_2_ electrodes as a function of incident light intensity and for various nitrogen doping levels. The inset presents characteristic photo-response curves for various illumination intensities from 20 to 66 µW·cm^−2^, for the sample N-TiO_2_-0.2. The current jump Δ*I* can be calculated from this data by subtracting the dark current from the saturation current as illustrated in the inset.

As the conductivity of semiconductors depends directly on the charge carrier density, it can be temporarily influenced by photo-generated electron-hole pairs into the material (*i.e.*, transitions of electrons from the valence band or donor levels to the conduction band). The technique is briefly presented in Supplementary materials. Considering the bandgap of anatase TiO_2_ [60,61], photo-excitation by wavelengths above 400 nm mainly involves electrons from the donor band while UV excitation mainly involves electrons initially in the valence band. Figure 8 presents the current change (ΔI), defined as the difference between the current level in the dark and the saturation value under illumination, as a function of UV light intensity (excitation in the 330–380 nm range, see the Experimental section) for the un-doped and N-doped porous electrodes deposited from the nanopowders. It is worth noting that a sintering procedure similar to that used for device fabrication was also carried out in this case.

In all cases, the current—and hence the conductivity—increases under illumination, and saturates after about 180 s (inset of Figure 8). All the nitrogen doped samples show similar photo-response curves. An increased nitrogen content in the film is associated with an increased current change. This behavior indicates that nitrogen doping increases the amount of charge carriers being promoted into the conduction band of TiO_2_, as also suggested by the TRMC analysis of the nanopowders and the intensities observed for the different doping levels (Figure 7). Unlike in our TRMC analysis, which showed faster charge recombination with doping (Figure 6a), the high voltage biasing of the devices used during the photo-conductivity measurements (bias voltage of 8 V) assists immediate charge transport to the electrodes preventing rapid charge recombination, so that a transient current signal can indeed be recorded. Thus, in this measurement, the limiting factor for the electrical response of the devices is not the charge recombination time but rather the density of recombination centers, e.g., impurities related to surface N atoms. The differences between the N-doped samples are small, as also noticed in the TRMC measurements made under UV excitation. A decrease in the photo-response is, however, observed for sample N-TiO2-0.6, which suggests that high nitrogen content is detrimental to current collection. This observation is consistent with the high density of surface nitrogen atoms revealed in this case, and with the high recombination rate evidenced for this sample in the previous section.

**Figure 9:**
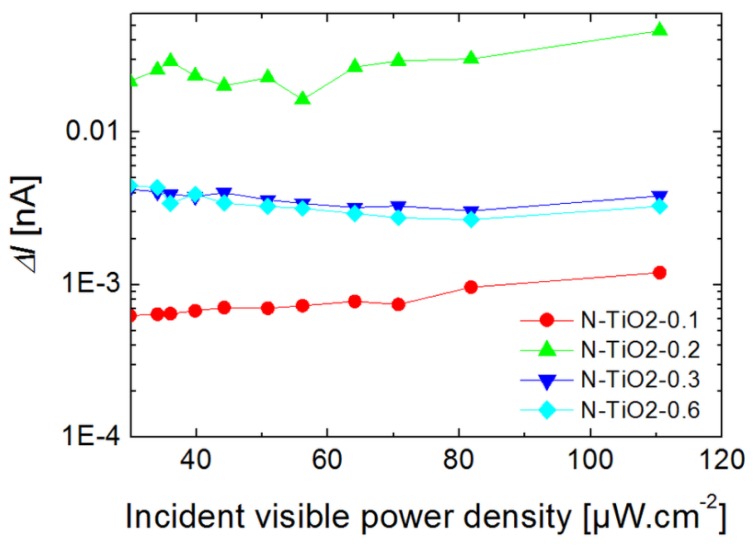
Current change associated to N-doped TiO_2_ porous films under visible light illumination as a function of incident light intensity.

Figure 9 presents the current change (ΔI) of N-doped films for an excitation in the visible range provided by a long-pass filtered halogen lamp (λ > 410 nm, see Experimental section), as a function of incident light intensity.

The un-doped film is not shown in Figure 9, as it is associated with a very low current level (before the light is switched on) and current change (<1 pA, see Supplementary materials, Figure S10) under illumination by visible light. This behavior is expected as the sample does not significantly absorb the incident light, so that no photo-conductivity is observable. On the opposite side, doped samples rapidly show a significant current change and photo-conductive behavior. We observe that after the initial jump corresponding to the beginning of the illumination, the current experiences a linear increase and no saturation can be observed. This linear increase, which was not observed under UV illumination, can be attributed to thermal effects induced by the infrared wavelengths of the light source. Filtering the infrared part of the lamp is indeed found to completely remove this linear dependency of the photo-response (see Supplementary materials, Figure S11). This effect is an illustration of the important temperature-dependency of $n_{d}$, and hence of ΔI.

Keeping this thermal effect in mind, we observe in Figure 9 a larger current level for porous films based on N-doped TiO_2_ compared to the un-doped reference. Both the dark current and current change ΔI under visible light are enhanced through doping (by about one order of magnitude). Similarly to previous sections, our data suggest that the photo-induced charge transport properties of the porous films are improved through nitrogen doping, especially within the visible range. 

## 3. Discussion

Nitrogen-doped porous electrodes can potentially improve the performance of dye-sensitized solar cells. However, although several reports discuss the positive effect of doping on device efficiency [24,32,33,34], it is also clear that true improvements remain subject to our ability to properly control both the amount and the location of the dopants. In the present study, as well as in our previous report [34], several sets of independent ssDSSC show that improvements in short-circuit current densities, open-circuit voltage, and in overall power conversion efficiencies can be achieved at low doping levels (Table 3). Our fabrication strategy exploits the laser pyrolysis process to grow TiO_2_ and N-doped TiO_2_ nanopowders of comparable morphologies and crystallinities (Table 1); however, nitrogen location, revealed by XPS analysis, is found to be strongly dependent on the doping level: although interstitial and substitutional N atoms are evident, the fraction of surface nitrogen increases with the doping level. While atoms inserted inside the TiO_2_ structure give the metal oxide a visible photo-activity, surface nitrogen can act as electron traps or recombination centers. The resulting surface states are found to be limiting the charge lifetime, as revealed by transient photo-voltage measurements performed at the device level (Figure 5), and by TRMC at the material level (Figure 6). Larger recombination rates are indeed observed for doped electrodes, as soon as nitrogen is incorporated in the material. In parallel, photo-conductivity measurements on porous films prepared from the synthesized TiO_2_ nanopowders (similar to device electrodes) show that better transport can be achieved in the doped electrodes, as long as recombination can be reduced through an external applied voltage for example (Figure 8). More specifically, our analysis gives direct evidence for the positive role played by the visible range photo-activity of the doped samples (both using TRMC and photo-response measurements). During illumination within the visible range, a larger fraction of charges can contribute to current collection, which is consistent with the signature observed in the IPCE spectra of the cells around 450 nm (Figure S7). However, in full working devices, such benefit of nitrogen doping remain subject to the competition with the intense recombination processes, which are found to mainly drive device operation. We strongly believe that the main limitation to current generation for the N-doped cells is associated with the preferential location of N atoms on the TiO_2_ particle surface. The XPS signatures of such type of nitrogen (Figure 3 and Table 2) start to be clearly observed for sample N-TiO_2_-0.2, when photocurrent also begins to drop. This drop is consistent with the spectral signature observed around 600 nm in the IPCE spectrum of the N-doped devices, which is associated with the influence of surface-related defects induced by the presence of N atoms. Such assumption was also proposed by Lindgren *et al.* for liquid DSSC, for example [28]. For higher doping level up to 0.6 wt %, when recombination is the main limiting factor, we observe a drastic drop of 25% in both photocurrent and device efficiency.

## 4. Materials and Methods 

### 4.1. Synthesis of N-Doped TiO_2_ Nanocrystals

Nanoparticles doped with different levels of nitrogen were synthesized through laser pyrolysis by varying the NH_3_ flow in the precursor mixture composed of titanium tetra-isopropoxide (TTIP) and C_2_H_4_. The details were reported in our previous work [36,38,62]. For the photovoltaic application, the as-prepared nanopowders were annealed at 400 °C for 3h to remove the free carbon phase that remains due to the decomposition of TTIP and/or C_2_H_4_. 

### 4.2. Device Fabrication

TiO_2_ and N-doped TiO_2_ porous electrodes were deposited on pre-cleaned FTO glass substrates initially covered with a TiO_2_ blocking layer deposited by chemical spray pyrolysis, using spin-coating from formulations based on ethanol, α-terpineol and ethyl-cellulose (EC), as previously described [38]. These films were progressively sintered up to 430 °C during 40 min in air. A TiCl_4_ treatment (Aldrich, 0.04 M in deionized water) was performed on these films before a final sintering step at 430 °C in air during 45 min. The 1.8 µm thick electrodes were then immersed in D102 dye (Mitsubishi Paper Mills, Tsukuba, Japan) dissolved in an acetonitrile:*tert*-butanol mixture (1:1 in volume) at 80 °C overnight. The sensitized electrodes were rinsed and infiltrated by the hole transporting material (HTM) spiro-OMeTAD (Merck KGaA, Darmstadt, Germany) by spin-coating in ambient conditions, following recipes previously reported [37,38]. Gold counter electrodes were evaporated through a shadow mask at 10^−6^ mbar, leading to two independent active areas of 0.18 cm² per cell.

### 4.3. Characterization Techniques

TEM images were recorded on a Philips CM12 microscope to examine the morphology of both the obtained nanoparticles and device cross sections. The crystalline phases were determined by XRD with a Siemens D5000 instrument using the Cu-Kα radiation. The specific surface of the nanopowders was determined by the BET method using a Micromeritics FlowsorbII 2300 instrument. The chemical environment of nitrogen atoms in TiO_2_ and N-TiO_2_ was characterized by X-ray photoelectron spectroscopy (XPS) using a Kratos Analytical Axis Ultra DLD spectrometer (Kα X-ray) on the powders. The optical properties of the powders were analyzed using a UV-visible-NIR spectrophotometer (Jasco V-570) in reflectance mode. According to the Kubelka–Munk equation, the optical gap can be estimated from the (F(*R*).hυ) plot as a function of photon energy, where F(*R*) = (1 − *R*)^2^/2*R* and *R* is the reflection coefficient [63]. The current density-voltage characteristics of the devices were recorded using a Keithley 2400 source-measure unit in the dark and under simulated solar emission (NEWPORT class A solar simulator) at 100 mW·cm^−2^ in AM1.5G conditions after spectral mismatch correction.

### 4.4. Transient Photo-Voltage Measurements

Transient photo-voltage (TPV) decay measurements were measured under open-circuit conditions using a set-up previously described [37]. Two continuous white LEDs (OSRAM) were used to provide the constant illumination of the device up to approximately 100 mW°C·cm^−2^. An additional pulsed LED (λ = 550 nm, Luxeon STAR, 5W), controlled by a solid state switch, generated a small light pulse on the cell. The transient charge population generated by this LED was always below a few percent of the continuous steady-state charge density in the device, ensuring a small-perturbation regime. The photo-voltage of the device was monitored and recorded using a digital oscilloscope (Tektronix DPO 4032) interfaced using a home-made Labview routine. The photo-voltage decays were adjusted using mono-exponential decay functions. 

### 4.5. Time-Resolved Microwave Conductivity Measurements (TRMC)

The charge-carrier lifetimes in the un-doped and N-doped TiO_2_ nanopowders after an illumination were determined by microwave absorption experiments using TRMC. The incident microwaves were generated by a Gunn diode of the Ka band at 30 GHz. The pulsed light source was an OPO laser from EKSPLA where the accord ability extends from 200 to 2000 nm. The full width at half-maximum of one pulse was 7 ns and the repetition rate of the experiments was 10 Hz. The light energy density received by the sample depends on the wavelength. To avoid the excitation energy effect on the signals, all the data are divided by the number of photons. Typically, the energy density at 450 nm is 5.2 × 10^−3^ J·cm^−2^, corresponding to 1.2 × 10^16^ photons·cm^−2^. 

### 4.6. Photo-Conductivity Measurements

Photo-conductivity characteristics of the samples were studied by illuminating un-doped and N-doped TiO_2_ thin films with UV (double peak between 330 and 380 nm, see Supplementary materials, Figure S12) and visible light (long-pass filtered halogen lamp, λ > 410 nm, see Supplementary materials, Figure S13) and measuring the photo-response of the conductivity of the film, *i.e.*, the change in the sample conductivity when turning on the light under a constant applied bias voltage. For these experiments, thin porous films were fabricated from the same TiO_2_ nanopowders used for the solar cell preparation, except that it was sonicated for 1h using a Hielscher sonicator (UP400s ultrasonicator, Hielscher Ultrasonics GmbH, Teltow, Germany) while cooled in a water path. After sonication, the powder was spin-coated on a glass chip (~1 cm^2^) at 1000 rpm for 1 min. To avoid cracks in the films, they were left to dry for 5 to 10 min before performing a first soft annealing step at 50 °C for 5 min in air. Then, the films were annealed at 430 °C for 35 min. Film deposition, as well as the drying and annealing steps, were performed in a laminar flow hood. Gold contact electrodes (100 nm thick) were evaporated under high vacuum using a thin copper wire (diameter ≤ 120 µm) placed as a mask across the film. For the photoconductivity measurements, samples were placed in a windowed vacuum chamber at 4–6 mbar to remove most of the ambient moisture. The response of photoconductivity as a function of illumination intensity was measured using Stanford Research Systems voltage and current low-noise preamplifiers (models SR560 and SR570). A MinUVIS 30 W mercury lamp (Desaga #751311 , Heidelberg, Germany) and a 50 W halogen lamp (Solux C5 12 V, Rochester, USA) were used for illumination, and light intensity was adjusted by changing the distance between the sample and the light source. The light intensities were measured using PD100M optical power meter (Thorlabs Sweden AB, Mölndal, Sweden) with S302C thermal power head (Thorlabs Sweden AB, Mölndal, Sweden).

## 5. Conclusions

TiO_2_ and N-doped TiO_2_ nanoparticles have been synthesized using laser pyrolysis, and used to deposit porous electrodes suitable for solid-state dye-sensitized solar cells. A systematic analysis of the physical properties of the samples as a function of doping level was performed in order to discuss the exact influence of the dopant on material and device photo-physics. At low doping levels, N atoms have been efficiently incorporated in interstitial positions into the metal oxide structure for annealed powders. In this case, device performance is found sensibly improved compared to pure TiO_2_. However, a large fraction of surface nitrogen is also observed at higher doping levels, which was found to be responsible for faster recombination kinetics that clearly reduces device efficiency. At the material scale, charge kinetics is found to be in good correlation with kinetics observed on devices. Both suggest faster electron-hole pair recombination being induced in the presence of N atoms compared to the un-doped powder. Our analysis confirms that the rapid drop in photocurrent observed in our ssDSSC at high doping levels can be associated with the preferential location of nitrogen atoms at the particle surface, which favor interfacial recombination. The visible photo-sensitivity of the samples is confirmed by TRMC measurements, which showed an increasing photo-conductivity of the material in visible range as the nitrogen content increases. This trend was further confirmed through photo-response measurement made on porous TiO_2_ and N-TiO_2_ films. Once again, the photo-conductivity of the material was found to be largely improved under visible excitation, compared to the pure TiO_2_ reference.

Although nitrogen doping remains a relevant strategy to improve the efficiencies of dye-sensitized solar cells, our data show that achieving concrete benefits at the device level from the visible photo-activity of N-doped TiO_2_ requires a fine control of the doping level and of the location of N atoms in the metal oxide structure. We also point out an interesting correlation between the photo-physical properties of samples probed at various scales from materials to devices. In light of our investigations, the specific features of the starting nanopowder materials are found to drive the device photo-physics, even when surface treatments and sintering steps are used during solar cell fabrication. 

## Figures and Tables

**Table 1:** Main physico-chemical properties of the TiO_2_ and N-doped TiO_2_ powders including N content determined by elemental analysis, anatase crystalline fraction and mean crystal diameter obtained by X-ray diffraction (XRD), as well as Brunauer, Emmett, and Teller (BET) specific area and mean grain diameter.

Sample	N content (wt %)	Data extracted from XRD		BET analysis	
		Fraction of anatase (%)	Mean crystal diameter (nm)	Specific area (m²·g^−1^)	Mean grain diameter (nm)
TiO_2_	<<0.1	94	15.6	77	20
N-TiO_2_-0.1	0.1	80	12.0	86	18
N-TiO_2_-0.2	0.2	90	11.4	86	18
N-TiO_2_-0.3	0.3	94	12.4	90	17
N-TiO_2_-0.6	0.6	94	15.0	96	16

**Table 2:** Relative contributions of the X-ray photoelectron spectroscopy (XPS) peaks observed for samples doped by nitrogen at various contents. The table is presenting data for annealed powders (see Supplementary materials for data associated with as-prepared samples).

Sample	Relative contributions of XPS features		
	Substitutional N (peak at 396 eV)	Interstitial N (peak at 400 eV)	Surface N (peak at 402 eV)
N-TiO_2_-0.1	11%	89%	Not measurable
N-TiO_2_-0.2	16%	74%	9%
N-TiO_2_-0.3	19%	58%	23%
N-TiO_2_-0.6	15%	46%	39%

**Table 3:** Photovoltaic parameters of solar cells based on N-doped TiO_2_ electrodes as a function of Nitrogen content.

Nature of porous electrode	V_OC_ (V)	J_SC_ (mA·cm^−2^)	FF	η (%)
TiO_2_	0.77	8.31	0.62	4.0
N-TiO_2_-0.1	0.79	8.31	0.62	4.1
N-TiO_2_-0.2	0.82	7.86	0.60	3.9
N-TiO_2_-0.3	0.77	7.00	0.61	3.3
N-TiO_2_-0.6	0.78	6.55	0.60	3.0

## Supplementary Materials

The following are available online at http://www.mdpi.com/2079-4991/6/3/35/s1. Complementary experimental details, Table S1: Experimental conditions of laser pyrolysis for the synthesis of N-doped TiO_2_ powders, Figure S1: XRD patterns of all powders, Figure S2: XPS spectra of TiO_2_ nanopowder, Figure S3: XPS spectra as-prepared N-TiO_2_-0.2 and N-TiO_2_-0.6 samples, Table S2: Relative contributions of XPS features for as-prepared N-doped samples, Figure S4: SEM cross-section micrographs of ssDSSC, Figure S5: Absorption coefficient of the dye-sensitized electrodes, Figure S6: J(V) curves of the solar cells in the dark, Figure S7: Normalized IPCE, Figure S8: Photoluminescence spectra of undoped and doped nanopowders, Figure S9: Normalized TRMC signals for different excitation wavelengths, Figure S10: Photoconductivity curves of porous films under visible light excitation, Figure S11: Photoconductivity curves of the porous film under visible light illumination, with and without infrared filter, Figure S12 : Emission spectrum of the UV-lamp used in the photoconductvity measurements, Figure S13: Optical spectrum of the long-pass filtered halogen lamp used in the photoconductivity measurements.
