# Supplementary material for: Influence of Nitrogen Doping on Device Operation for TiO2-Based Solid-State Dye-Sensitized Solar Cells: Photo-Physics from Materials to Devices
Source: Nanomaterials (Basel). 2016 Feb 23;6(3):35. doi: 10.3390/nano6030035 (PMC5302520; doi:10.3390/nano6030035)
Supplement: Supplementary file 1 [file nanomaterials-06-00035-s001.pdf]

Supplementary Materials: Influence of Nitrogen Doping on Device Operation for TiO_2-based Solid-State Dye-Sensitized Solar Cells: Photo-Physics from Materials to Devices

Jin Wang, Kosti Tapio, Aurélie Habert, Sebastien Sorgues, Christophe Colbeau-Justin, Bernard Ratier, Monica Scarisoreanu, Jussi Toppari, Nathalie Herlin-Boime and Johann Bouclé

Complementary Experimental Details

Chemical composition of the powders. The carbon and nitrogen contents were measured using two elemental analysis instruments (Carbon/Sulfur Analyzer EMIA-320V Series, Horiba, and Oxygen/Nitrogen/Hydrogen Analyzer EMA-800 series, respectively).

Optical absorption of the dye-sensitized electrodes. The optical properties of the dye-sensitized electrodes were measured in transmission mode by a UV-visible spectrometer SAFAS DES 200.

IPCE spectra of ssDSSC devices. The incident photon to charge carrier efficiency spectra were measured in static regime using a monochromated 75 W Xenon lamp (Newport) and a calibrated picoamperemeter (Keithley 485). A certified silicon photodiode of known spectral response was used for the calibration of the incident photon flux.

Photoluminescence spectra of the un-doped and N-doped nanopowders. Steady-state photoluminescence spectra were probed by a FLS980 spectrometer (Edinburgh Instruments). The excitation was performed using a Xenon lamp and a monochromator and the detection was made between 200 and 870 nm by a R928P Hamamatsu photomultiplier (cooled, dark count <50 cps). The powders were placed in a quartz sample holder and monochromator slits were adjusted to lead to a bandwidth of around 1.5 nm. All the spectra are normalized with regard to the emission pic at 3.15 eV, which is attributed to the free exciton emission of anatase TiO_2.

TRMC experiments. The TRMC technique is based on the measurement of the time evolution of the microwave power $\Delta P(t)$ reflected by a sample, following an excitation pulse delivered by a laser [1,2]. For small perturbations, the relative variation of the reflected microwave power $\Delta P(t)/P$ can be correlated with the variation of conductivity $\Delta\sigma(t)$ of the probed material as:

$$\frac{\Delta P(t)}{t} = A\Delta\sigma(t) = Ae\sum_i \Delta n_i(t)\cdot\mu_i \tag{1}$$

where, $\Delta n_i(t)$ is the number of excess charge carriers of species i at time t, and μ_i is the corresponding charge mobility. The sensitivity factor A is independent of time, but depends on different factors such as the microwave frequency or the dielectric constant of the materials studied. Considering that the mobility of trapped species is usually several orders of magnitude lower than that of mobile charge carriers, and considering the n-type behavior of TiO_2 materials, $\Delta n_i(t)$ reduces to mobile electrons in the conduction band of the metal oxide [3].

Photoconductivity measurements. In a constant-voltage photo-response measurement, the change in conductivity $\Delta\sigma$ due to generation of electron-hole pairs by a constant light intensity E_e will be observed as a current jump, ΔI, which will depend on the density of photo-generated charge carriers n_c promoted into the conduction band of TiO_2. The change of conductivity, and thus the change of current, can be written as:

$$\Delta I = V\Delta\sigma = Ve\mu_e\Delta n_c \tag{2}$$

where e is the elementary charge, μ_e the electron mobility, V the bias voltage, and Δn_c the difference between the number of conduction band electrons in the dark and under illumination. As the occupation of the valence band n_v does not depend strongly on temperature, the UV response is stable against small temperature fluctuations. On contrary, larger temperature dependency is expected when the excitation is made in the visible range since in this case the valence band electrons can be neglected, and ΔI can be approximated as:

$$\Delta I \cong Ve\mu_e E_e n_d = Ve\mu_e E_e e^{\frac{-E_d}{k_b T}} \quad (3)$$

where E_d and n_d are the energy and the occupation of the donor band, T the temperature, and k_b Boltzmann's constant.

Custom-made vacuum chamber with transparent window was used in photo-voltage measurements. Visible and infrared measurements were carried out using 50 W halogen lamp (Solux C5 12 V) and UV measurements using 30 W Hg low-pressure lamp (Desaga MinUvis).

Table S1. Experimental conditions of laser pyrolysis for the synthesis of N-doped TiO_2 powders and corresponding fraction of nitrogen and carbon, as determined by elemental analysis.

Sample	NH_3 Flow ($cm^3 \cdot min^{-1}$)	As-prepared powders		Annealed powders	
		wt % N	wt % C	wt % N	wt % C *
TiO_2	0	<0.1	1.23	<0.1	0.14
N-TiO_2-0.1	96	0.9	2.69	0.1	0.16
N-TiO_2-0.2	170	1.1	2.85	0.2	0.18
N-TiO_2-0.3	325	2.0	2.77	0.3	0.18
N-TiO_2-0.6	424	2.5	3.14	0.6	0.21

Notes: * The C content of a commercial TiO_2 sample (TiO_2 P25) was also measured for reference and the measured value was 0.15 wt %, similar to that of our annealed powders, indicating that this value is consistent with samples free of significant carbon phases.

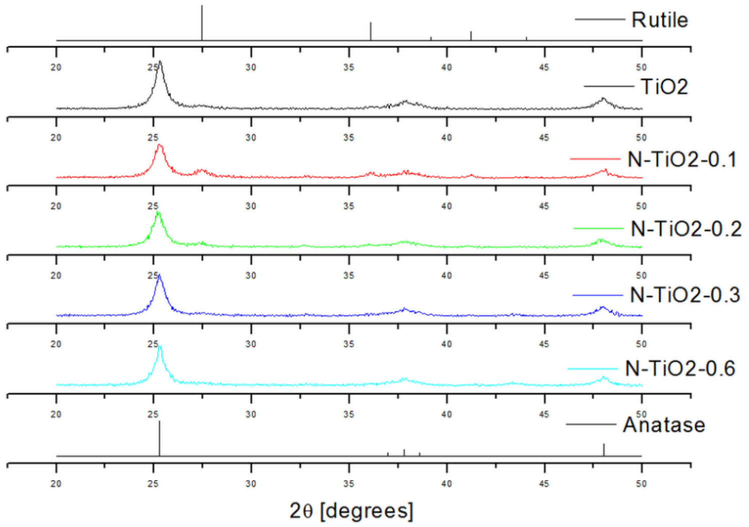

Figure S1. XRD patterns of all powders.

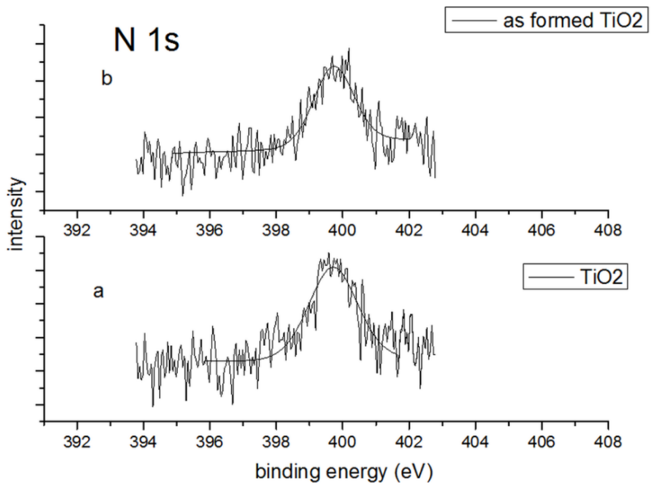

Figure S2. XPS spectra of (**a**) as-prepared and (**b**) annealed pure TiO_2 nanopowder.

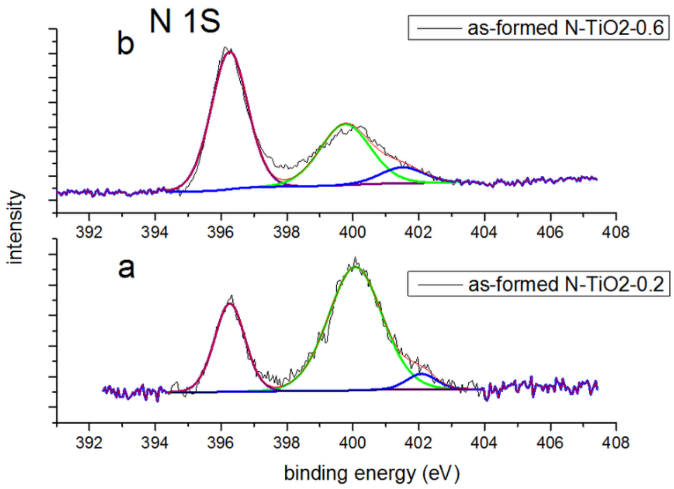

Figure S3. XPS spectra of as-prepared (**a**) N-TiO_2-0.2 and (**b**) N-TiO_2-0.6 samples.

Table S2. Relative contributions of XPS features for as-prepared N-doped samples N-TiO_2-0.2 (N content of 0.2 wt %) and N-TiO_2-0.6 (N content of 0.6 wt %).

Sample	Relative contributions of XPS features		
	Substitutional N (peak at 396 eV)	Interstitial N (peak at 400 eV)	Surface N (peak at 402 eV)
As-prepared N-TiO_2-0.2	27%	68%	5%
As-prepared N-TiO_2-0.6	57%	35%	8%

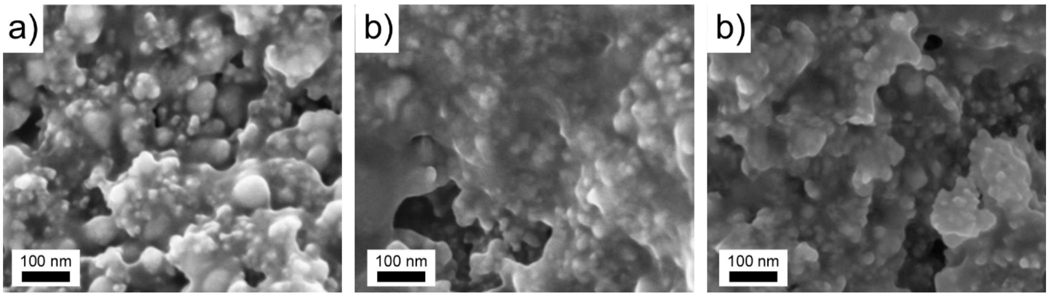

Figure S4. SEM cross-section micrographs of ssDSSC based on (**a**) pure TiO_2, (**b**) N-TiO_2-0.2, and (**c**) N-TiO_2-0.6 porous electrodes.

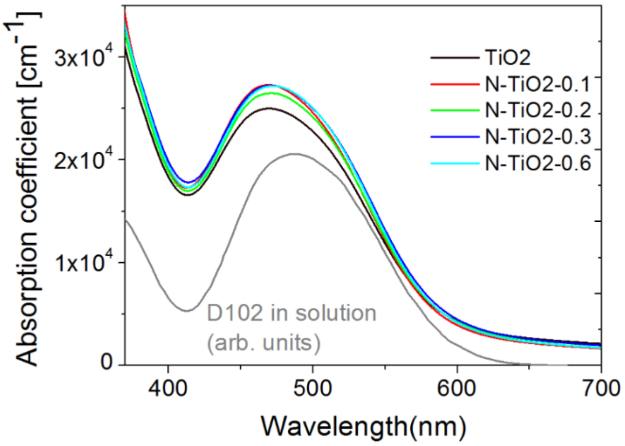

Figure S5. Absorption coefficient of the dye-sensitized electrodes, before the infiltration of the HTM, for several N-doping levels. The absorption coefficient of the D102 dye in solution is given for reference (grey line).

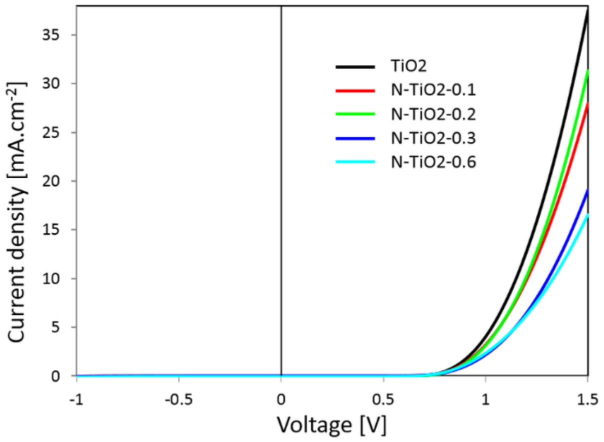

Figure S6. J(V) curves of the solar cells in the dark for several doping levels.

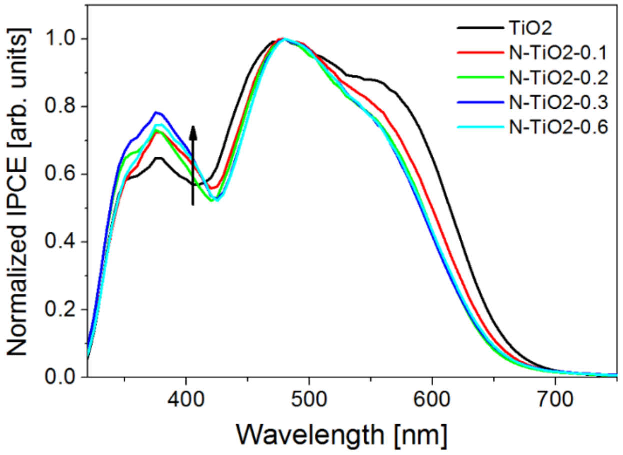

Figure S7. Normalized IPCE of the ssDSSC as a function of N-doping level.

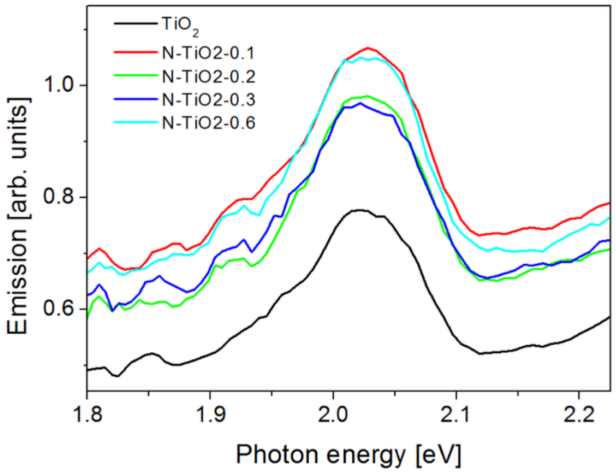

Figure S8. Photoluminescence spectra of the pure and N-doped TiO_2 nanopowders. The excitation is performed at 300 nm.

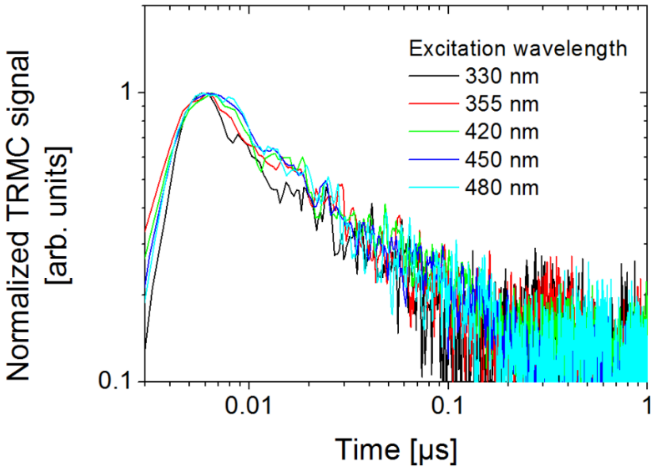

Figure S9. Normalized TRMC signals of the N-doped nanopowders sample N-TiO2-0.6 (N content of 0.6 wt %) for different excitation wavelengths.

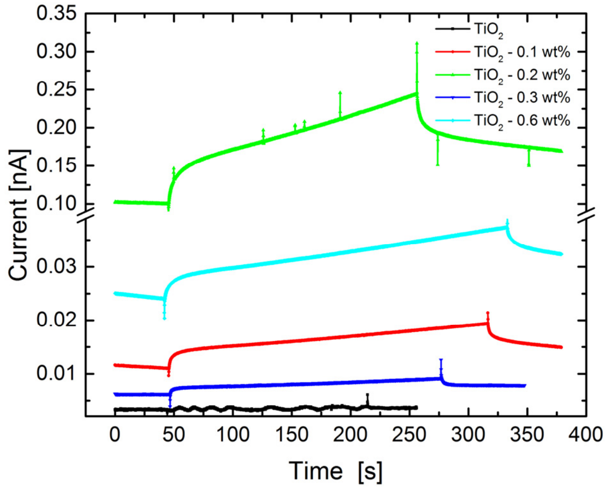

Figure S10. Photoconductivity curves of un-doped and N-doped TiO2 porous films under visible light excitation at 66 μW·cm^{-2}. All of the doped samples show at least a small linear response under illumination due to heating (Infrared filter was not used), whereas the current of the undoped sample only oscillates.

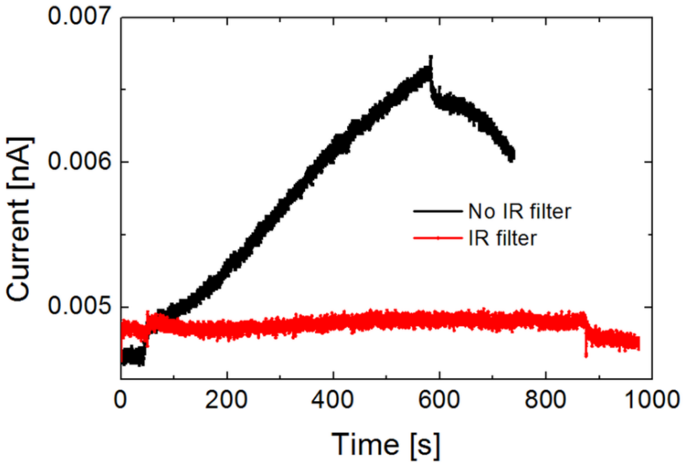

Figure S11. Photoconductivity curves of the porous film prepared from sample N-TiO2-0.2 under visible light illumination, with and without infrared filter (water filter).

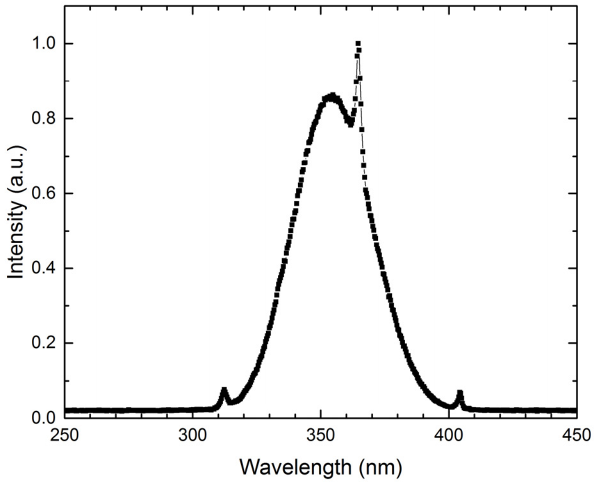

Figure S12. Emission spectrum of the UV-lamp used in the photoconductivity measurements.

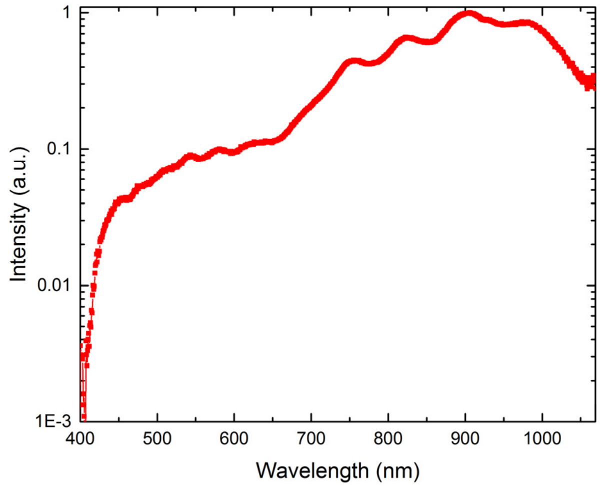

Figure S13. Optical spectrum of the long-pass filtered halogen lamp used in the photoconductivity measurements.

References

1. Martin, S.T.; Herrmann, H.; Choi, W.; Hoffmann, M.R. Time-resolved microwave conductivity. Part 1.—TiO_2 photoreactivity and size quantization. *Faraday Trans. J. Chem. Soc.* **1994**, *90*, 3315–3322.
2. Martin, S.T.; Herrmann, H.; Hoffmann, M.R. Time-resolved microwave conductivity. Part 2.—Quantum-sized TiO_2 and the effect of adsorbates and light intensity on charge-carrier dynamics. *Faraday Trans. J. Chem. Soc.* **1994**, *90*, 3323–3330.
3. Colbeau-Justin, C.; Kunst, M.; Huguenin, D. Structural influence on charge-carrier lifetimes in TiO2 powders studied by microwave absorption. *J. Mater. Sci.* **2003**, *38*, 2429–2437.

© 2016 by the authors; licensee MDPI, Basel, Switzerland. This article is an open access article distributed under the terms and conditions of the Creative Commons by Attribution (CC-BY) license (http://creativecommons.org/licenses/by/4.0/).
